# Sensitive Albumin Test

**Published:** 1913-12

**Authors:** 


SENSITIVE ALBUMIN TEST. Five c.c. of filtered urine is put into each of three test tubes. To Nos. 1 and 2 is added one c.c. of diluted acetic acid; then to No. 1 is added 5 c.c. of the following reagent:

| | |
|---|---|
| Mercuric chloride.................... | 10 |
| Citric acid............................ | 20 |
| Sodium chloride...................... | 20 |
| Distilled water........................ | 200 |

Tubes 2 and 3 are then filled up with water to the same height as No. 1. All three are shaken and examined against a dark background; a cloudiness in No. 1 indicates albumin; in No. 2 mucin or nucleo-albumin, the latter being confirmed by an increased cloudiness on adding water. Alkaline urine should first be acidified with nitric acid. It is stated that one part of albumin in 120,000 can be detected by this test.—Jolle: *Allgemeine Wiener Med. Zeit.,* October, 1912, per *Universal Med. Record.*

